# Modular Assembly of Ordered Hydrophilic Proteins Improve Salinity Tolerance in *Escherichia coli*

**DOI:** 10.3390/ijms22094482

**Published:** 2021-04-25

**Authors:** Leizhou Guo, Mingming Zhao, Yin Tang, Jiahui Han, Yuan Gui, Jiaming Ge, Shijie Jiang, Qilin Dai, Wei Zhang, Min Lin, Zhengfu Zhou, Jin Wang

**Affiliations:** 1 College of Life Science and Engineering, Southwest University of Science and Technology, Mianyang 621000, China; guoleizhou102819@163.com (L.G.); tangyin15280939267@163.com (Y.T.); gui_yuan2021@163.com (Y.G.); sjjiang0406@swust.edu.cn (S.J.); daiqilinmj@sina.com (Q.D.); 2 Biotechnology Research Institute, Chinese Academy of Agricultural Sciences, Beijing 100081, China; 82101182064@caas.cn (M.Z.); 13121257599@163.com (J.H.); gejiaming98@163.com (J.G.); zhangwei01@caas.cn (W.Z.); linmin57@vip.163.com (M.L.)

**Keywords:** hydrophilic protein, modular assembly, order, salinity tolerance

## Abstract

Most late embryogenesis abundant group 3 (G3LEA) proteins are highly hydrophilic and disordered, which can be transformed into ordered α-helices to play an important role in responding to diverse stresses in numerous organisms. Unlike most G3LEA proteins, DosH derived from *Dinococcus radiodurans* is a naturally ordered G3LEA protein, and previous studies have found that the N-terminal domain (position 1–103) of DosH protein is the key region for its folding into an ordered secondary structure. Synthetic biology provides the possibility for artificial assembling ordered G3LEA proteins or their analogues. In this report, we used the N-terminal domain of DosH protein as module A (named DS) and the hydrophilic domains (DrHD, BnHD, CeHD, and YlHD) of G3LEA protein from different sources as module B, and artificially assembled four non-natural hydrophilic proteins, named DS + DrHD, DS + BnHD, DS + CeHD, and DS + YlHD, respectively. Circular dichroism showed that the four hydrophile proteins were highly ordered proteins, in which the α-helix contents were DS + DrHD (56.1%), DS + BnHD (53.7%), DS + CeHD (49.1%), and DS + YLHD (64.6%), respectively. Phenotypic analysis showed that the survival rate of recombinant *Escherichia coli* containing ordered hydrophilic protein was more than 10% after 4 h treatment with 1.5 M NaCl, which was much higher than that of the control group. Meanwhile, in vivo enzyme activity results showed that they had higher activities of superoxide dismutase, catalase, lactate dehydrogenase and less malondialdehyde production. Based on these results, the N-terminal domain of DosH protein can be applied in synthetic biology due to the fact that it can change the order of hydrophilic domains, thus increasing stress resistance.

## 1. Introduction

Abiotic stresses, such as drought, radiation, oxidation, and high or low temperatures, can promote organisms to produce reactive oxygen species (ROS). Excessive ROS not only causes DNA double-strand breaks, as well as lipid, protein, and membrane damage, but it also affects the expression of some related genes in biological metabolic pathways, and ultimately affects growth [1,2]. In the process of evolution, organisms have formed unique protection mechanisms in response to adversity stress to adapt to unfavorable environments [3]. The synthesis of hydrophilic protein is one of the important regulatory mechanisms for organisms to respond to abiotic stress and reduce cell damage. Late Embryogenesis Abundant proteins (LEA) are a large class of hydrophilic proteins that play an important role in plant drought resistance and other abiotic stress tolerance [4,5].

The group 3 LEA (G3LEA) proteins are the most widely studied among the LEA family proteins and highly hydrophilic, which originally were found in plants and later were found in other species, such as bacteria[6], mosses [7], and even some invertebrates [8]. G3LEA proteins have anti-aggregation, stable proteins and chaperone-like activities [4,9]. When G3LEA proteins are used as hydrophilic proteins, they can protect cell metabolism and reduce damage caused by the external environment [10]; as chaperone-like, they have the effects of binding ions and preventing oxidation [11,12,13]. In vitro enzyme protection assays have also confirmed that G3LEA proteins can protect lactate dehydrogenase (LDH) from freezing damage [14] and protect citrate synthase (CS) and malate dehydrogenase(MDH) from drought damage [15]. Overexpression of G3LEA proteins can improve the dehydration, cold tolerance, and high salinity tolerance of recombinant *E. coli*, transgenic rice, and wheat [16,17].

The occurrence of a highly conserved 11-mer repeating motif (TAQAAKEKAGE) in the primary sequence is a major characteristic of G3LEA proteins [18]. This motif is characterized by apolar residues in positions 1, 2, 5, and 9, and charged or amide residues in positions 3, 6, 7, 8, and 11 [19,20]. An α helical arrangement of the 11-mer repeating unit gives an amphiphilic helix whose hydrophobic stripe twists in a right-handed fashion around the helix [19]. The number and size of motifs in G3LEA proteins vary by species; for example, the soybean PM2 protein contains 6 repeats [18], the *C.elegans* protein contains 24 repeats, and cotton D-29 protein contains 21 repeats [19]. The fragment composed of the 11-mer motifs of G3LEA proteins is also called the hydrophilic domain (HD) [21]. Liu et al. selected four HDs from *D. radiodurans*, *Caenorhabditis elegans*, *Yarrowia lipolytica* and *Brassica napus* due to their sequence similarity, comparatively conserved HDs and their representativeness, and they found that heterologous HD expression confers enhanced drying and oxidation tolerance to *E. coli* [21]. Based on their results, the HDs of G3LEA proteins are potential ideal stress-response elements that can be applied in synthetic biology due to their extraordinary protection and stress resistance ability.

At present, it is known that most G3LEA proteins and their hydrophilic domains (HD) are intrinsically disordered proteins (IDPs) in aqueous solutions, but, when the solution contains SDS, TFE, glycerol, methanol, or is under drought conditions, they become highly ordered α-helix structure [22,23,24]. The DosH (*Deinococcus* ordered stress-inducible Hydrophilic) protein derived from *D. radiodurans* belongs to the G3LEA protein family, and its secondary structure determination shows that it contains 45% α-helix, which is a naturally ordered G3LEA protein and involved in the cellular defense against oxidative stress [25]. Previous research in our laboratory found that the N-terminal domain (position 1–103) of the DosH protein alone does not function, but it is the key region that helps the DosH protein fold into an ordered secondary structure (data not shown). Synthetic biologists have taken inspiration from natural protein networks to assemble different protein modules to create new functional proteins [26]. Based on these concepts, we want to use synthetic biology methods to reassemble protein modules from different sources to construct new ordered hydrophilic proteins and explore whether ordered hydrophilic proteins can enhance the stress resistance of organisms.

In this study, the N-terminal domain of DosH protein was used as module A and named DS, and four hydrophilic domains (DrHD, BnHD, CeHD, and YlHD) of G3LEA proteins which have been characterized were used as module B [21]. Module A and module B were reassembled to form new hydrophilic proteins, named DS + DrHD, DS + BnHD, DS + CeHD, and DS + YlHD, respectively. We first analyzed the structure of these hydrophilic proteins by circular dichroism and identified their possible functions by heterologous expression in *Escherichia coli*. This research focuses on the potential use of synthetic biology to manipulate the structure of hydrophilic proteins involved in cellular stress tolerance, which is novel and provides ideas for the design of artificial stress elements in synthetic biology.

## 2. Results

### 2.1. Silico Analysis of Four Hydrophilic Proteins

The full length of DosH protein contains 298 amino acids, and the relative molecular weight is 30.93 KDa (Figure S1A). Based on previous reports, we divided its structure into a conserved N-terminal domain (position 1–103), a hydrophilic domain (HD, position 104–263), and a short-terminal peptide C-terminal (position 264–298) (Figure S1B). Prediction by IUPred showed that the order of DosH protein may be 26.2%, and the ordered regions could be mainly distributed at the N-terminal, when the protein is in aqueous solution (Figure S1C). The N-terminal of DosH protein contained 103 amino acids with a relative molecular weight of 10.86 KDa, which was rich in amino acid A (18.4%), K (10.7%), L (9.7%), and G (9.7%) (Figure S1D). NCBI multiple sequence alignment analysis showed that the N-terminal domain was unique and highly conserved in the genus *Deinococcus*, especially the regions 10–82 and 87–101, and we named it DS (Figure S2).

According to the modular assembly principle of proteins in synthetic biology, the DS domain (module A) and the hydrophilic domain of G3LEA protein (from other species, as module B) can be assembled into a non-natural protein. In this study, we assembled four novel non-natural proteins, named DS + DrHD, DS + BnHD, DS + CeHD, and DS + YLHD, and these hydrophilic domains were derived from *D. radiodurans*, *B. napus*, *C. elegans* and *Y. lipolytica*, respectively (Figure S1E). ProtParam and Kyte and Doolittle online predicted that the hydrophobic indexes of DS + DrHD, DS + BnHD, DS + CeHD, and DS + YLHD were −0.744, −0.969, −1.128, and −1.030, respectively, indicating that the four non-natural proteins were highly hydrophilic (Figure S3, Table S1), so they were also called hydrophilic proteins in this study.

The structural order predicted for DS + DrHD, DS + BnHD, DS + CeHD, and DS + YlHD was 31.5%, 23.3%, 18.4%, and 23.9%, respectively. At the same time, the structural order predicted for BnHD, DrHD, CeHD, and YlHD was 0%, 2.5%, 4.8%, and 13.7%, respectively, which theoretically indicated that four non-natural hydrophilic proteins could be more ordered.

### 2.2. Order Analysis of Four Non-Natural Hydrophilic Proteins

We constructed recombinant vectors combining the DS domain of DosH protein with four hydrophilic domains, and then transformed them into *E. coli* BL21 (DE3) to induce expression and purification of the proteins. The proteins were analyzed by far-UV circular dichroism (CD) spectroscopy and recombinant proteins were diluted to 0.2 mg mL^−1^ in 10 mM phosphate buffer (pH 7.0).

**Figure 1:**
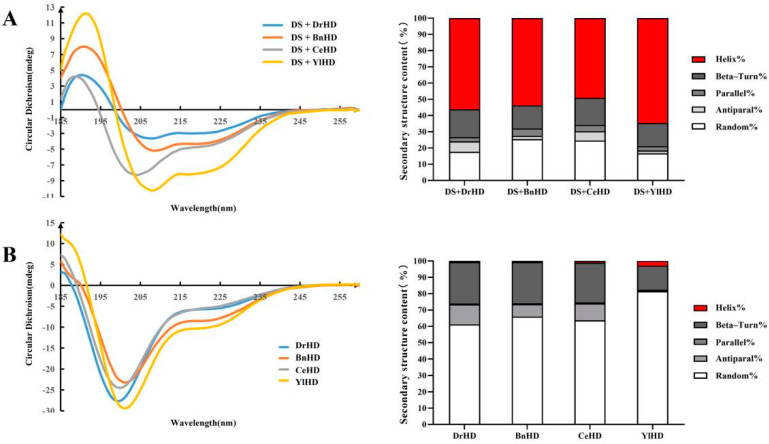
CD analysis of hydrophilic proteins. (**A**) The secondary structure of four non-natural hydrophilic proteins (**B**) and hydrophilic domain proteins in phosphate buffer. Percentages of a random coil (white), beta-Turn (dark grey), parallel (grey), antiparallel (light grey), and α-helix (red) are deduced from CD spectra using the CDNN program.

The secondary structure determination showed that the recombinant proteins DS + DrHD, DS + BnHD, DS + CeHD, and DS + YlHD were formed positive peaks near 190 nm, and negative peaks appeared near 222 nm and 208 nm (Figure 1A, left), which were typical α-helix structures, indicating that the four non-natural hydrophilic proteins were highly ordered. The secondary structure content of each hydrophilic protein in a normal phosphate buffer was shown on the right side of Figure 1A. The ordered indices of DS + DrHD, DS + BnHD, DS + CeHD, and DS + YlHD were 82.3%, 74.5%, 75.4%, and 83.3%, respectively, of which their α-helix contents were 56.1%, 53.7%, 49.1%, and 64.6%, respectively (Table S2). In addition, the proteins DrHD, BnHD, CeHD, and YlHD had obvious negative peaks at 200 nm in normal phosphate buffer, indicating that they were typical disordered structures and the α-helix content of all hydrophilic domains was less than 3% (Figure 1B, Table S2).

### 2.3. Ordered Hydrophilic Proteins Improved Salinity Tolerance of E. coli

**Figure 2:**
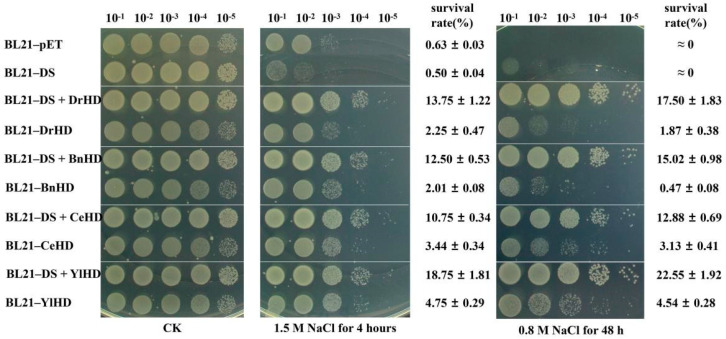
Survival phenotype plate assay of *E. coli* recombinant strains under high salt condition.

To explore whether modularly assembled ordered hydrophilic proteins could improve the stress resistance of *E. coli*, we treated the recombinant strains with abiotic stresses, such as oxidation, desiccation, and high salt, and the results showed that they had great sensitivity difference to high salt. As shown in Figure 2, the recombinant strains containing ordered hydrophilic proteins had higher resistance than BL21-pET, BL21-DS, and four strains containing hydrophilic domain after treatment for 4 h in LB medium containing 1.5 M NaCl, and their survival rate exceeded 10% (the middle of Figure 2). When we spot the recombinant strains directly on the solid medium containing 0.8 M NaCl, the phenotypic results became more obvious (the right of Figure 2). The survival rate of recombinant strains containing ordered hydrophilic proteins was more than 12%, and BL21-DS + YLHD reached 22.55%. The empty strain BL21-pET and the recombinant strain BL21-DS containing the N-terminal domain of the DosH protein could not survive in this environment, indicating that the DS module alone does not play a role in improving the salinity tolerance of *E. coli*.

After two different salt stress treatment strips, the recombinant strains containing hydrophilic domain also showed some salt resistance, and their survival rate, from high to low, was as follows: BL21-YlHD > BL21-CeHD > BL21-DrHD > BL21-BnHD.

### 2.4. Ordered Hydrophilic Proteins Increased the Total Antioxidant Capacity of E. coli

**Figure 3:**
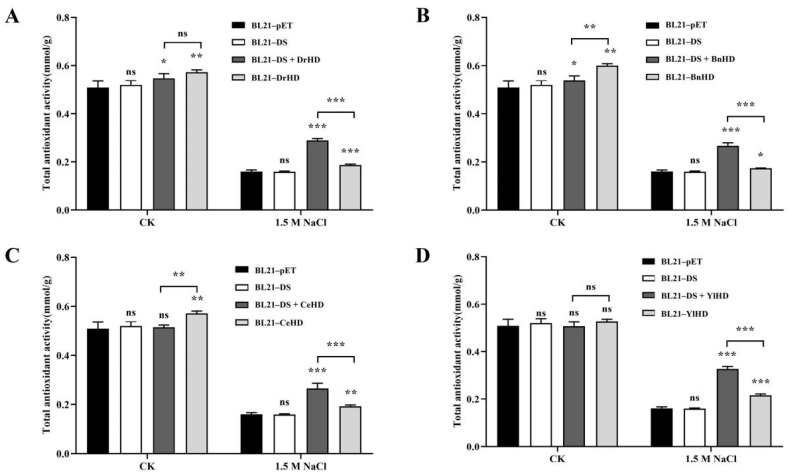
Total antioxidant capacity analysis. (**A**) Analysis of total antioxidant capacity of recombinant strains BL21-DS + DrHD and BL21-DrHD. (**B**) Analysis of total antioxidant capacity of recombinant strains BL21-DS + BnHD and BL21-BnHD. (**C**) Analysis of total antioxidant capacity of recombinant strains BL21-DS + CeHD and BL21-CeHD. (**D**) Analysis of total antioxidant capacity of recombinant strains BL21-DS + YlHD and BL21-YlHD. The symbols ‘ns’, ‘*’, ‘**’ and ‘***’, respectively, represent ‘no significantly different (*p* > 0.05)’, ‘a significant difference (0.01 < *p* < 0.05)’, ‘an extremely significant difference (0.001 < *p* < 0.01)’, and ‘the most significant difference (*p* < 0.001)’.

Cells will produce reactive oxygen species during normal physiological metabolism. At the same time, some environmental factors, such as ultraviolet radiation, strong osmotic pressure, high or low temperature, and oxidation, can also induce the production of reactive oxygen species [27]. There are a variety of antioxidants in cells, including antioxidant macromolecules, antioxidant small molecules and enzymes, which can remove reactive oxygen species in cells. The total level of various antioxidant macromolecules, antioxidant small molecules and enzymes in a system reflects the total antioxidant capacity of the system. Under normal growth conditions, the recombinant strains BL21-BnHD and BL21-CeHD had higher total antioxidant capacity than the recombinant strains BL21-DS + BnHD and BL21-DS + CeHD (Figure 3B,C), but, after treatment with 1.5 M NaCl for 4 h, the total antioxidant capacity of the four recombinant strains containing ordered hydrophilic protein was higher than that of the recombinant strain BL21-DS. In addition, the total antioxidant capacity of each recombinant strain containing ordered hydrophilic proteins was higher than that of the recombinant strain containing hydrophilic domains of the same origin.

BL21-DrHD, BL21-BnHD, BL21-CeHD, and BL21-YlHD also had higher total antioxidant capacity than the empty strain BL21-pET.

### 2.5. E. coli Containing Ordered Hydrophilic Protein Had Higher SOD and CAT Activity

Active oxygen is a kind of oxygen-containing compound with strong oxidizing ability produced by aerobic metabolism of organisms [28], including O_2_^−^, OH and H_2_O_2_, etc. [29]. Several enzyme systems catalyze reactions to neutralize free radicals and active oxygen species. These enzymes include superoxide dismutase (SOD), catalases (CAT), peroxidase, and glutathione reductase [30].

**Figure 4:**
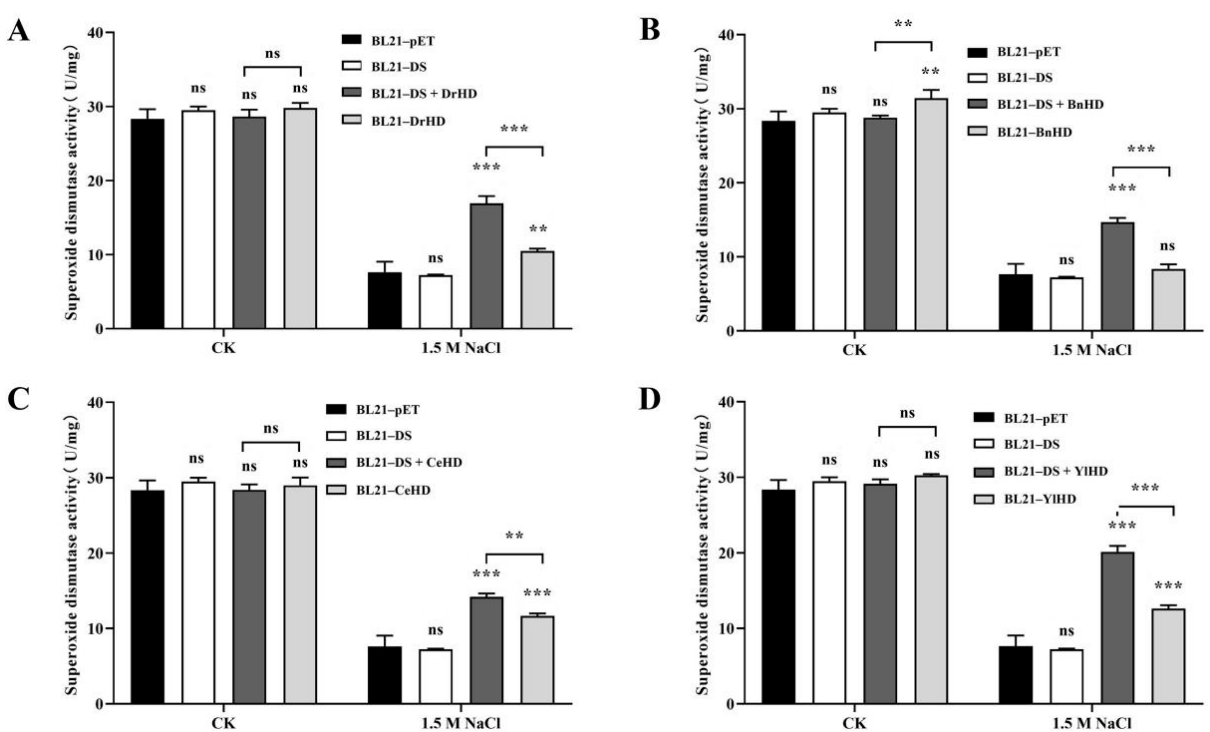
Superoxide dismutase activity analysis. (**A**) Analysis of superoxide dismutase activity of recombinant strains BL21-DS + DrHD and BL21-DrHD. (**B**) Analysis of superoxide dismutase activity of recombinant strains BL21-DS + BnHD and BL21-BnHD. (**C**) Analysis of superoxide dismutase activity of recombinant strains BL21-DS + CeHD and BL21-CeHD. (**D**) Analysis of superoxide dismutase activity of recombinant strains BL21-DS + YlHD and BL21-YlHD. The symbols ‘ns’, ‘**’ and ‘***’, respectively, represent ‘no significantly different (*p* > 0.05)’, ‘a significant difference (0.01 < *p* < 0.05)’, ‘an extremely significant difference (0.001 < *p* < 0.01)’, and ‘the most significant difference (*p* < 0.001)’.

Under normal culture conditions, among the 4 groups of samples, only BL21-BnHD has higher SOD activity than BL21-DS + BnHD (Figure 4B). After treatment with 1.5 M NaCl for 4 h, the enzyme activity values of all recombinant strains containing ordered hydrophilic protein were significantly higher than those of BL21-DS, BL21-pET, and strains containing only HD (Figure 4). The results of CAT activity in vivo showed that, under normal culture conditions, recombinant strains BL21-DS + DrHD, BL21-DS + BnHD, BL21-DS + CeHD, and BL21-DS + YlHD had higher enzyme activity, which was also significantly higher than those of BL21-DS and recombinant strains containing only hydrophilic domains (Figure S4). After treatment with 1.5 M NaCl for 4 h, all the recombinant strains with DS domain still had the highest enzyme activity. These results suggest that non-natural ordered hydrophilic proteins might better protect SOD and CAT when *E. coli* was tolerant to high salt stress.

### 2.6. E. coli Containing Ordered Hydrophilic Protein Had Higher LDH Activity and Less MDA Production

**Figure 5:**
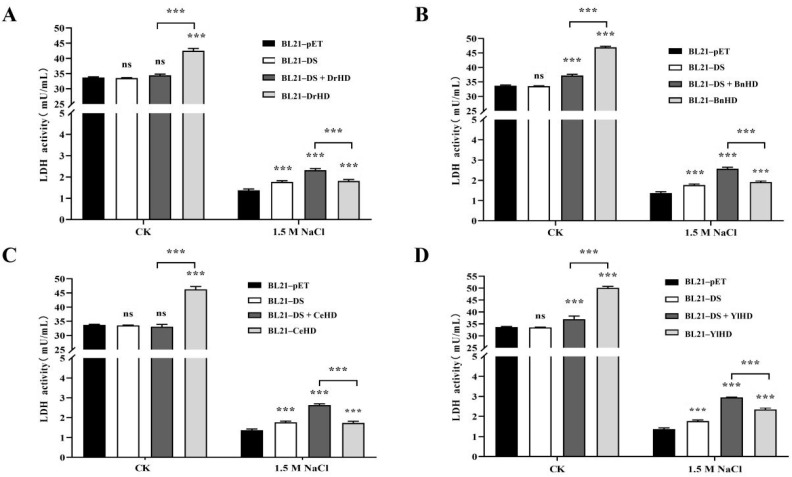
Lactate dehydrogenase activity analysis. (**A**) Analysis of lactate dehydrogenase activity of recombinant strains BL21-DS + DrHD and BL21-DrHD. (**B**) Analysis of lactate dehydrogenase activity of recombinant strains BL21-DS + BnHD and BL21-BnHD. (**C**) Analysis of lactate dehydrogenase activity of recombinant strains BL21-DS + CeHD and BL21-CeHD. (**D**) Analysis of lactate dehydrogenase activity of recombinant strains BL21-DS + YlHD and BL21-YlHD. The symbols ‘ns’ and ‘***’, respectively, represent ‘no significantly different (*p* > 0.05)’, ‘a significant difference (0.01 < *p* < 0.05)’, ‘an extremely significant difference (0.001 < *p* < 0.01)’, and ‘the most significant difference (*p* < 0.001)’.

Studies have shown that G3LEA proteins and their hydrophilic domains perform multiple functions in an ordered structure, such as chaperone-like, which can protect enzymes (lactate dehydrogenase, malate dehydrogenase, citrate synthase, etc.) or proteins (α-casein) from damage under stress [31]. Lactate dehydrogenase (LDH) enzyme is very stable under normal conditions but tends to be inactivated under freezing, drought, and high-temperature conditions [4]. At present, LDH is used as model enzymes to detect the protective function of proteins on enzymes. In vivo LDH activity assay results (Figure 5) showed that, under normal conditions, all recombinant strains containing hydrophilic domains had higher enzyme activity than recombinant strains BL21-DS and BL21-PET, as well as recombinant strains containing ordered hydrophilic proteins. After treatment with 1.5 M NaCl for 4 h, the LDH enzyme activity of all strains decreased, but the recombinant strains containing ordered hydrophilic proteins had relatively high enzyme activity values, indicating that the unnatural ordered hydrophilic proteins might be involved in protecting LDH from high cell osmotic pressure and reduced its aggregation in *E. coli*.

**Figure 6:**
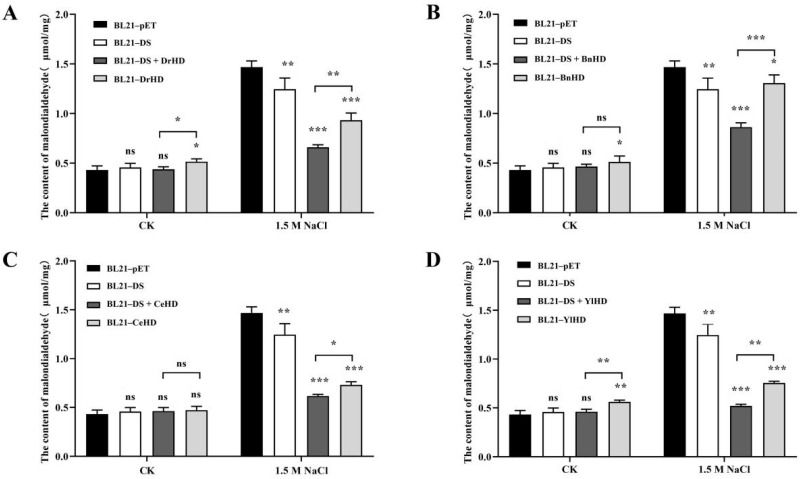
Analysis of MDA production. (**A**) Analysis of MDA production of recombinant strains BL21-DS + DrHD and BL21-DrHD. (**B**) Analysis of MDA production of recombinant strains BL21-DS + BnHD and BL21-BnHD. (**C**) Analysis of MDA production of recombinant strains BL21-DS + CeHD and BL21-CeHD. (**D**) Analysis of MDA production of recombinant strains BL21-DS + YlHD and BL21-YlHD. The symbols ‘ns’, ‘*’, ‘**’ and ‘***’, respectively, represent ‘no significantly different (*p* > 0.05)’, ‘a significant difference (0.01 < *p* < 0.05)’, ‘an extremely significant difference (0.001 < *p* < 0.01)’, and ‘the most significant difference (*p* < 0.001)’.

Malondialdehyde (MDA) is a lipid peroxide formed by oxygen free radicals in cells that attack polyunsaturated fatty acids in biological membranes. The content of MDA can reflect the degree of lipid peroxidation, and indirectly reflect the degree of cell damage [32]. After treatment with 1.5 M NaCl for 4 h, the results of MDA content determination (Figure 6) showed that all recombinant strains containing the N terminal of DosH protein domain produced less MDA than those containing only HD domain, indicating that ordered hydrophilic proteins had a certain protective ability to the cell membrane of *E. coli* in a high salt environment and could reduce cell membrane damage.

## 3. Discussion

Abiotic or environmental stresses, such as high soil salinity, extreme temperature, water deficiency, and unsuitable pH, are major limiting factors for the growth and productivity of all living organisms [33]. The group 3 LEA (G3LEA) proteins are well-characterized hydrophilic proteins that upregulate in response to environmental stresses, such as desiccation, freezing, and high salinity [34,35], and they are described not only throughout the plant kingdom but also in other organisms, ranging from invertebrates to prokaryotes [36].

G3LEA proteins are also intrinsically disordered proteins (IDPs) [34]: they are disordered in the hydrated state but become more ordered upon dehydration when they predominantly form α-helical structures [8,22,37]. The 11-mer motifs region constitutes the hydrophilic domain of the G3LEA protein. Hydrophilic domains derived from nematodes, *D. radiodurans*, rape, and yeast can form highly ordered α-helical structures under the induction of glycerol or TFE [21]. Nuclear magnetic resonance (NMR) analysis on the sequence of the motif composed of 11 amino acids from the soybean G3LEA protein (PM2) showed that the motif was disordered in aqueous solution, but, after the addition of SDS, it became an α-helix structure [18]. We have summarized the related work that has been done to determine the order of G3LEA proteins (Table S5). Except for the DrLEA3 protein (also called DosH protein) derived from *D. radiodurans* having 45% α-helix in its natural state, the order of G3LEA proteins from other sources does not exceed 10% α-helical and becomes more orderly only with SDS, TFE, or drying process (Table S3). The high α-helix of DosH protein has aroused our research interest. Our previous research has found that the high α-helix of DosH protein is inseparable from its N-terminal (data not shown). The results of this article also confirm our previous data. The α-helix of the recombinant proteins DS + DrHD (lack C-terminal domain compared to DosH protein) and DrHD (the hydrophilic domain of DosH protein) are 56.1% and 0.7%, respectively, suggesting that the N-terminal domain plays an important role in the folding of Dosh into an ordered secondary structure. After recombining the DS domain of DosH protein with hydrophilic domains from other sources, the α-helix content of all reassembled hydrophilic proteins increased significantly, indicating that they are all ordered proteins, which is consistent with the trend predicted by IUPred. The specific mechanism by which the addition of the DS domain increases the order of the hydrophilic domains of G3LEA protein is currently unknown.

It has been reported that the N-terminal domain may play an important role in the spatial folding of LEA protein. Previous results suggest that the N-terminal domain of group 1 LEA proteins may be important for proper folding during dehydration [38]. Karamjeet et al. predicted the conserved domains of plant G3LEA proteins and found that there are four N-terminal conserved motifs (called MAaRS, MARS, MGRX, and M [AS] [RK], respectively), which may be the signal for group 3 protein to locate mitochondria [39]. At LEA4-5, a member of group 4 of LEAs in *Arabidopsis*, it has been found that their N-terminal region can undergo a transition to a partially folded state and prevent enzyme inactivation, and, interestingly, the ability to gain structure under water limiting conditions is limited to the conserved region at the N-terminal, indicating the N-terminal could function as a chaperone [40,41]. The late embryogenesis abundant (LEA)-like protein CDeT11-24 of *C. Plantagineum* carries an N-terminal lysine-rich sequence, which is identified this region to be responsible for both activities: enzyme protection and phosphatidic acid interaction [42]. The DS domain of DosH protein is different from the above-mentioned LEA protein N-terminal, and it is a specific N-terminal domain of *Deinococcus* (Figure S2), indicating that it might be the result of horizontal gene transfer. The high conservation of the DS domain of DosH protein also implied that it played an important function in the abiotic stress of *Deinococcus*, and whether the conserved DS domains in other *Dinococcus* can also increase the order of hydrophilic domains remains to be confirmed. Most classifications of LEA proteins are based on sequence similarity, protein domains, motif and composition [4,19,20]. Although the DosH protein has several tandem repeats of loosely conserved 11-mer motifs [34], it is different from the traditional G3LEA proteins, which is a naturally ordered G3LEA protein. Based on the above description, DosH might be classified as a new G3LEA protein subfamily.

The analysis of amino acid composition (Table S1) showed that the addition of the DS domain of DosH protein increased the number of charged amino acid residues (15 positively charged residues and 16 negatively charged residues) and hydrophobicity of each recombinant protein (Figure S3, Table S1). The charge number and hydrophobicity of the protein are the main factors affecting the spatial structure. Positively and negatively charged side chains have the tendency to attract each other; side chains with identical charges repel each other [43]. The bonds formed by the forces between the negatively charged side chains of aspartic or glutamic acid, on the one hand, and the positively charged side chains of lysine or arginine, on the other hand, are called salt bridges [44]. The hydrophobic interaction is that the nonpolar valine, leucine, isoleucine, and phenylalanine cause the displacement of water molecules [45]. In summary, the introduction of the DS domain of the DosH protein may affect the spatial structure by increasing the charge number and hydrophobicity of the recombinant protein. The amino acid composition results showed that only the N-terminal domain contains H, P, I, and Y, which might be closely related to the function of the DosH protein (Figure S1D).

Several mechanisms have been proposed for the function of G3LEA proteins: cytoskeleton formation [46], molecular shielding [47], ion sequestration [19], and vitrification [37]. Among them, the molecular shielding mechanism may best explain the anti-aggregation effect of G3LEA proteins: they act as a physical barrier between target biological molecules and, thereby, decrease the collision frequency of potentially aggregating species in cells [47]. Essentially, the protective effect of the hydrophilic domains is the same as that of the G3LEA proteins, with molecular shielding or chaperone-like activity [23,47]. Previous studies have confirmed that the hydrophilic domains directly interact with LDH to inhibit the aggregation and protect its activity through microscale thermophoresis experiments [21]. In vivo enzyme activity assay results showed that, after 4 h of treatment with 1.5 M NaCl, the recombinant strains containing ordered hydrophilic protein had a higher protective ability of LDH, revealing that the artificially ordered G3LEA proteins might better play the role of molecular shielding or chaperone-like. Paramagnetic quenching NMR experiments reveal the orientation of the 11-mer motif relative to the simulated membrane, in which the ordered N-terminal fragments are inserted into the simulated membrane, while the disordered C-terminal fragments are exposed to water, suggesting that the 11-mer motif may play an important role in the membrane stabilization of G3LEA proteins [18]. LEAM protein derived from pea (*Pisum sativum*) belongs to the G3LEA family, which interacts with the membrane in the dry state and protects the liposomes subjected to drying [22]. The DosH protein has also been confirmed to be enriched on the cell membrane and bind to a variety of metal ions in vivo [25]. These results indicate that the G3LEA proteins may be involved in the stability of cell membrane. In this research, we measured the MDA content of the recombinant strains before and after high salt treatment, and the results showed that the recombinant strains with DS domain produced less MDA, which, to some extent, also indicated that ordered hydrophilic proteins might protect cell membranes from lipid oxidative damage through some mechanism.

Synthetic biology has become a research focus in recent years. Modern synthetic biology has been transformed into an engineering discipline to design new organisms and better understand basic biological mechanisms [48]. The synthetic biology toolkit includes various programmable DNA [49], RNA [50], and protein regulatory elements [51]. Many of these biochemical elements are derived from natural biological elements, but some are completely synthetic [48]. In vitro experiments have long proved that the G3LEA protein and its hydrophilic domain function as ordered α-helices, but there is no direct evidence in vivo. The use of synthetic biology to artificially construct ordered hydrophilic proteins (or modules) provides the possibility to explain the correlation between the order of hydrophilic domains and the anti-stress function. Studies have shown that the four HDs (DrHD, BnHD, CeHD, YlHD) are expected to act as protective agents against desiccation and oxidative stress, are ideal stress response elements, and can be used in synthetic biology [21]. In our study, after the recombination of the N-terminal domain (as module A) of the DosH protein and the hydrophilic domains (as module B) from different organisms, the order of the unnatural hydrophilic proteins was significantly increased, and the salt tolerance of *E. coli* was improved. Phenotypic results on the surface of solid LB medium containing 0.8 M NaCl showed that the addition of DS had a difference in improving the salt tolerance of the recombinant strain compared with the strain containing HD only, which may be because of their characteristics are not well understood or well-matched between different modules. The survival difference between recombinant strain BL21-DS + YlHD and BL21-YlHD was the largest, followed by recombinant strain BL21-DS + DrHD and BL21-DrHD, while the survival difference between recombinant strain BL21-DS + CeHD and BL21-CeHD was the smallest. These results showed that the matching degree between DS and 4 different HDs from high to low was: DS + YlHD > DS + DrHD > DS + BnHD > DS + CeHD. It is not difficult to understand the high matching between DS and DrHD, but the high matching between DS and YlHD might be that DrHD and YlHD have relatively high evolutionary affinity (Figure S5).

## 4. Materials and Methods

### 4.1. Strains, Plasmids and Culture Conditions

The strains and plasmids used in this study are described in Table S4. *Escherichia coli* strains were cultured at 37 °C in Luria-Bertani (LB) medium (Tryptone 10 g/L, Yeast Extract 5 g/L, NaCl 10 g/L, pH 7.0, solid medium with 1.5% agar) or on LB plates with appropriate antibiotics as required.

### 4.2. Construction of Recombinant Vector and E. coli Strains

*DrHD*, *BnHD*, *CeHD*, and *YlHD* were amplified from the genomes of *Deinococcus radiodurans*, *Brassica napus*, *Caenorhabditis elegans*, and *Yarrowia lipolytica*, respectively. The N-terminal *ds* sequence of DosH protein was obtained from the genome of *Deinococcus radiodurans*. The amplified fragments were homologously recombined with the pET28a plasmid (digested with *BamH* I and *Hind* III) through Vazyme C115 CloneExpress^®^ Ultra One Step Cloning Kit to obtain *pET28a-DS + DrHD*, *pET28a-DrHD*, *pET28a-DS + BnHD*, *pET28a-BnHD*, *pET28a-DS + CeHD*, *pET28a-CeHD*, *pET28a-DS + YlHD*, *pET28a-YlHD*, and *pET28a-DS* recombinant plasmids. All recombinant plasmids and an empty pET28a vector were introduced into host strain *E. coli* BL21(DE3) to generate the transformed strains BL21-pET, BL21-DS + DrHD, BL21-DrHD, BL21-DS + BnHD, BL21-BnHD, BL21-DS + CeHD, BL21-CeHD, BL21-DS + YlHD, BL21-YlHD, and BL21-DS. All specific primers in the experiment (Table S5) were synthesized by BGI Company (Beijing, China).

### 4.3. Circular Dichroism (CD) Spectroscopic Analysis

Circular dichroism is a spectroscopic method based on the light absorption process, and it is the most widely used method for determining the secondary structure of proteins [52]. It can sensitively detect changes in protein conformation in a physiological solution. *Escherichia coli* BL21(DE3) strains carrying recombinant plasmids, named BL21-DS + DrHD, BL21-DrHD, BL21-DS + BnHD, BL21-BnHD, BL21-DS + CeHD, BL21-CeHD, BL21-DS + YlHD, and BL21-YlHD, were grown in LB medium supplemented with 50 μg mL^−1^ kanamycin at 37 °C until OD_600_ = 0.6 was reached and then induced with 0.1 mM isopropyl β-D-1-thiogalactopyranoside (IPTG) at 37 °C for 4 h. The cells were harvested, and the pelleted cells were suspended in a phosphate buffer. Cells were lysed by sonication on ice and then centrifuged (14,000× *g* at 4 °C for 15 min). Affinity chromatography with Ni-NTA Agarose (Cat#R90115; Invitrogen, New York, NY, USA) was used for purification, and refer to Liu et al. for detailed methods [21]. The secondary structure changes of purified recombinant proteins were measured by ChirascanTM circular bichromatic spectrometer (Applied Photophysics, Leatherhead, UK). CDNN software was used to analyze the secondary structure relative content of all recombinant proteins.

### 4.4. Salt Stress Tolerance in E. coli

The recombinant strain was transferred to 20 mL fresh LB medium according to OD_600_ = 0.1 (containing 50 mg/mL Kan) and cultivated to OD_600_ = 0.6 at 37 °C. The experiment was divided into two conditions: the first one is to resuspend the strains in LB liquid medium containing a final concentration of 1.5 M NaCl for 4 h, and then immediately use phosphate buffer for a 10-fold dilution (10^−1^ to 10^−^^5^), take 8 μL dots on the surface of LB solid medium and incubate at 37 °C for about 24 h to observe the growth. The second method is to directly dilute the recombinant strain by 10-fold dilution (10^−1^ to 10^−^^5^), take 8 μL of sample spot on the surface of LB solid medium containing different concentrations of NaCl, and incubate at 37 °C for about 48 h to observe the growth. The control group did not contain NaCl or NaCl treatment, and all experiments were repeated at least three times. The survival rate was expressed as the percentage of the number of colonies in the treated samples compared with that in the untreated *E. coli* sample used as a control.

### 4.5. In Vivo Enzyme Activity Assay

The recombinant strain was transferred to 20 mL fresh LB medium according to OD_600_ = 0.1 (containing 50 mg/mL Kan) and cultivated to OD_600_ = 0.6 at 37 °C. We added NaCl with a final concentration of 1.5 M for 4 h, centrifuged at 5000 rpm for 5 min, washed the cells three times with phosphate buffer (pH = 7.5), and, finally, vortexed it in 5 mL fresh phosphate buffer for ultrasonic disruption. After the broken cells were centrifuged at 12,000 rpm for 30 min, the supernatant was collected in a new centrifuge tube, and the protein concentration was determined using the Bradford method.

Refer to the Total Antioxidant Capacity Assay Kit with FRAP method (Cat#S0116; Beyotime Institute of Biotechnology, Shanghai, China) to detect the total antioxidant capacity [53], the Total Superoxide Dismutase Assay Kit with WST-8 (Cat#S0101M; Beyotime Institute of Biotechnology, Shanghai, China) to detect superoxide dismutase activity and the Catalase Assay Kit (Cat#S0051; Beyotime Institute of Biotechnology, Shanghai, China) to detect catalase [54,55]. Refer to the Lactate Dehydrogenase Activity Assay Kit (Cat#MAK066-KT; Sigma-Aldrich, Lenexa, KS, USA) to detect lactate dehydrogenase activity and refer to Lipid Peroxidation MDA Assay Kit (Cat#S0131S; Beyotime Institute of Biotechnology, Shanghai, China) to detect malondialdehyde [53,56].

## 5. Conclusions

In conclusion, based on the modular assembly principle of synthetic biology proteins, four novel non-natural hydrophilic proteins were reassembled from the N-terminal domain of DosH protein and the hydrophilic domain of G3LEA protein from different sources. The reassembled hydrophilic proteins show a highly ordered secondary structure. Phenotypic analysis of *Escherichia coli* showed that the recombinant strain containing ordered hydrophilic protein had higher salt tolerance. In addition, in vivo enzyme activity assay showed that the recombinant *Escherichia coli* containing ordered hydrophilic protein had higher activities of SOD, POD, and LDH and less MDA production than the control group after treatment with 1.5 M NaCl for 4 h, indicating that ordered hydrophilic protein plays an important role in physiological metabolism of *Escherichia coli* to tolerate high salt environment. Based on these results, the N-terminal domain of DosH protein is expected to be a new stress-response element. We can assemble the N-terminal domain of DosH protein and the hydrophilic domain of other G3LEA proteins into a new module by using synthetic biology methods to increase abiotic stress resistance by improving the order of the hydrophilic domain.

## Data Availability

All data underlying the results are included as part of the published article and its Supplementary Materials.

## Supplementary Materials

The following are available online at https://www.mdpi.com/article/10.3390/ijms22094482/s1, Figure S1: Analysis of the order and amino acid composition of the N-terminal domain of DosH protein; Figure S2: Multiple sequence alignment analysis based on DosH protein in *Deinococcus*; Figure S3: Hydropathic index plot of recombinant proteins analyzed by using the Kyte-Doolittle algorithm; Figure S4: Catalase activity analysis; Figure S5: Phylogenetic analysis of hydrophilic domains from different organisms; Table S1: The composition of each recombinant protein analyzed by using the ProtParam; Table S2: Secondary structure content in eight recombinant proteins was obtained by far UV CD spectrometry and calculated with CDNN software; Table S3: The characteristics of G3LEA proteins reported; Table S4: Strains and plasmids used in this study; Table S5: List of primers required for construction of recombinant *E. coli* strain. References [8,22,24,25,37,57,58,59,60,61,62,63] are cited in the supplementary materials.
