# Supplementary material for: Modular Assembly of Ordered Hydrophilic Proteins Improve Salinity Tolerance in Escherichia coli
Source: Int J Mol Sci. 2021 Apr 25;22(9):4482. doi: 10.3390/ijms22094482 (PMC8123400; doi:10.3390/ijms22094482)
Supplement: Supplementary file 1 [file ijms-22-04482-s001.zip › ijms-1165579-supplementary.pdf]

Modular Assembly of Ordered Hydrophilic Proteins Improve Salinity Tolerance in *Escherichia coli*

Leizhou Guo[1], Mingming Zhao[2], Yin Tang[1, 2], Jiahui Han[2], Yuan Gui[1, 2], Jiaming Ge[2], Shijie Jiang[1], Qilin Dai[1], Wei Zhang[2], Min Lin[2] , Zhengfu Zhou[2,*] and Jin Wang[1,2,*]

1 College of Life Science and Engineering, Southwest University of Science and Technology, Mianyang 621000, Sichuan, China; guoleizhou102819@163.com (L.G.); tangyin15280939267@163.com (Y.T.); 583224795@qq.com (Y.G.); daiqilinmj@sina.com (Q.D.); sjjiang0406@swust.edu.cn (S.J.);

2 Biotechnology Research Institute, Chinese Academy of Agricultural Sciences, Beijing 100081, China; 82101182064@caas.cn (M.Z.); 13121257599@163.com (J.H.); 741032147@qq.com (J.G.); zhangwei01@caas.cn (W.Z.); linmin57@vip.163.com (M.L.)

* Correspondence: zhouzhengfu@caas.cn (Z.Z.); wangjin@caas.cn (J.W.);

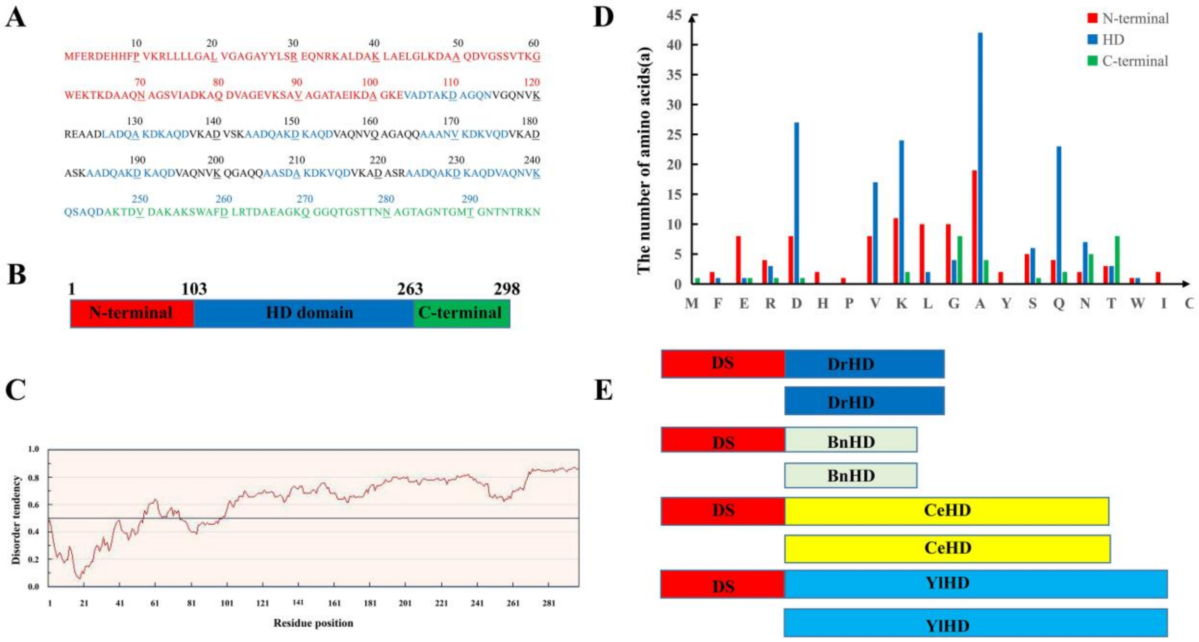

Figure S1. Analysis of the order and amino acid composition of the N-terminal domain of DosH protein. (A) The full-length sequence of DosH protein. The N-terminal domain (red), 8 motifs (blue), the C-terminal domain (green); (B) Model diagram of DosH protein; (C) Analysis of the order of the DosH protein; (D) Amino acid composition of DosH protein N-terminal domain (DS), hydrophilic domains (HD) and C-terminal domain, different alphabet represent different amino acids; (E) The model of recombinant protein construction.

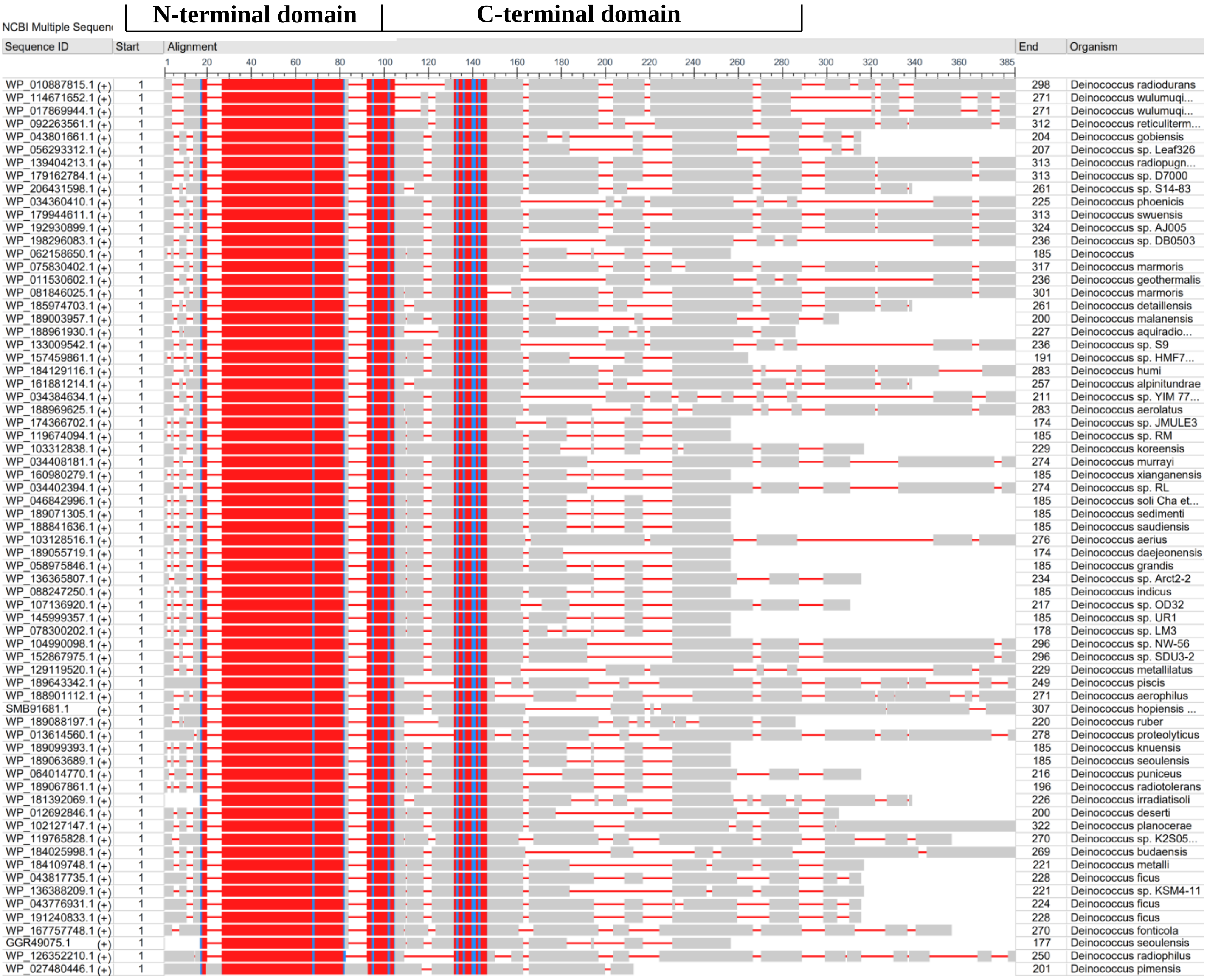

Figure S2. Multiple sequence alignment analysis based on DosH protein in *Deinococcus*. This method highlights highly conserved and less conserved amino acid positions based on the relative entropy threshold of the residue. Only alignment positions with no gaps will be colored. Red indicates highly conserved positions and blue indicates lower conservation.

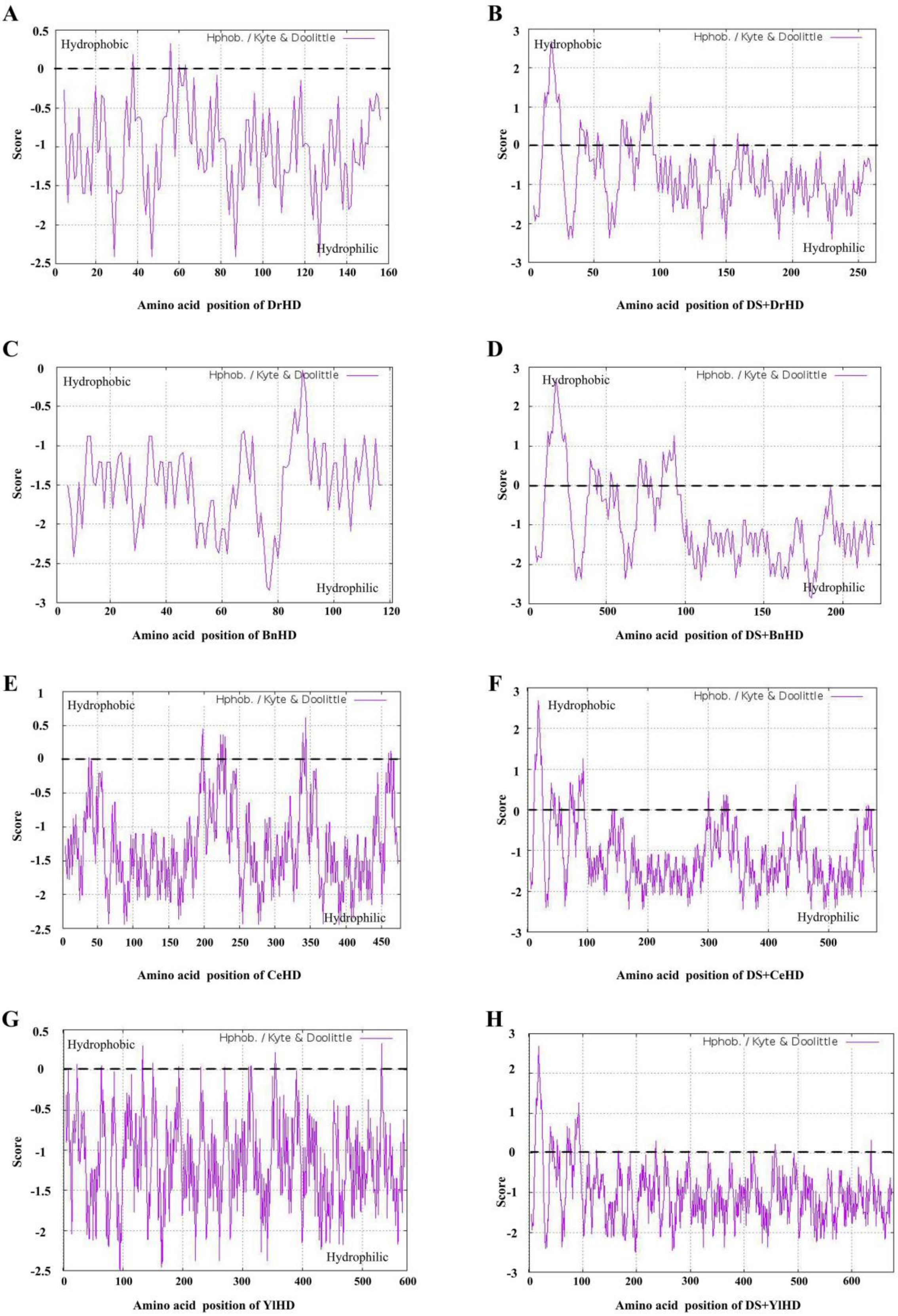

Figure S3. Hydropathic index plot of recombinant proteins analyzed by using the Kyte-Doolittle algorithm. Regions with a hydropathy score below zero are hydrophilic. A, C, E and G are the hydropathic predictions of proteins DS+DrHD, DS+BnHD, DS+CeHD and DS+YlHD; B, D, F and H are the hydrophilic and hydrophobic predictions of the proteins DrHD, BnHD, CeHD and YlHD;.

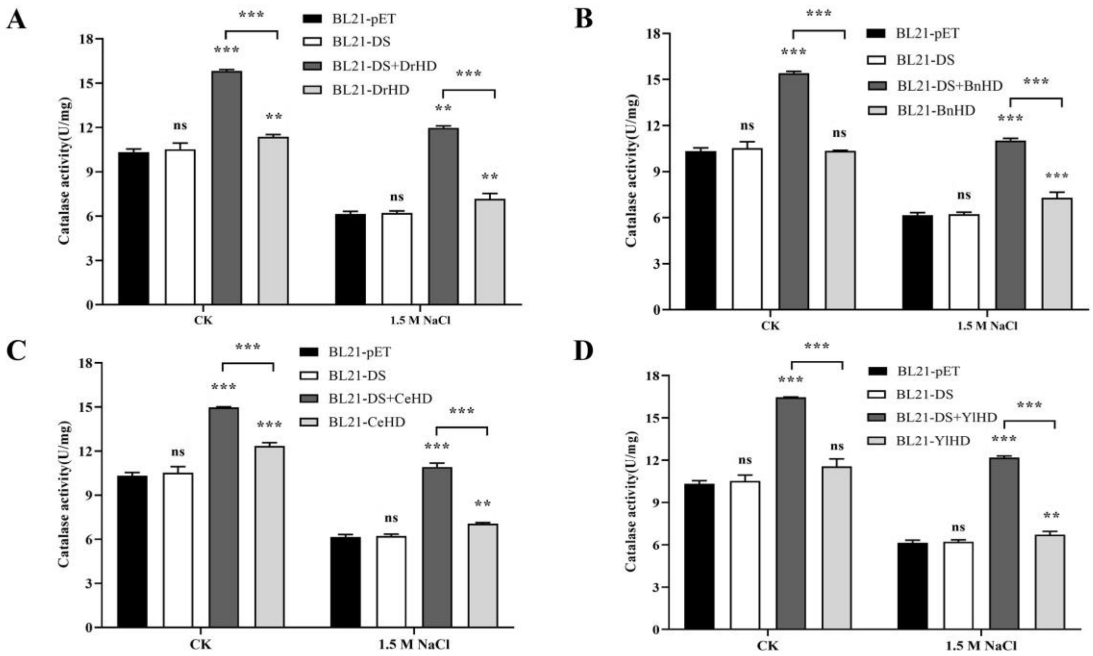

Figure. S4. Catalase activity analysis**.** The symbols ‘ns’, ‘*’, ‘**’ and ‘***’ respectively represent ‘no significantly different (p>0.05)’, ‘a significant difference (0.01<p<0.05)’, ‘an extremely significant difference (0.001<p<0.01)’ and ‘the most significant difference (p<0.001)’.

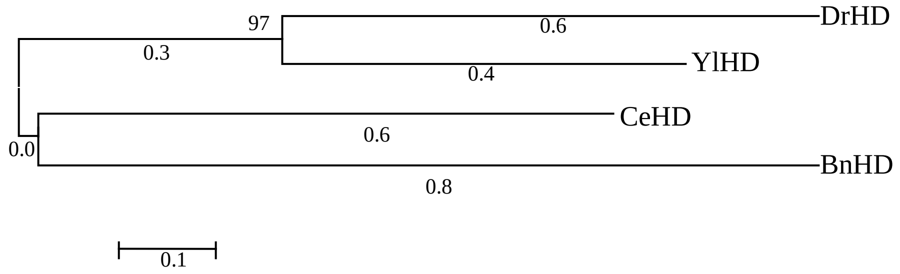

Figure. S5. Phylogenetic analysis of hydrophilic domains from different organisms. The tree was created using MEGA6.06 with 1000 bootstrap replicates. Bar: 0.1 substitutions per amino acid.

Table S1 The composition of each recombinant protein analyzed by using the ProtParam

Protein	Theoretical pI	Total number of positively charged residues (Arg + Lys)	Total number of negatively charged residues (Asp + Glu)	Instability index	Aliphatic index	Grand average of hydropathicity (GRAVY)	Formula	Molecular weight
DS	6.10	15	16	15.85	86.41	–0.361	$C_{475}H_{773}N_{137}O_{152}S_1$	10867.25
DS+DrHD	5.84	42	44	16.59	71.52	–0.744	$C_{1170}H_{1926}N_{360}O_{406}S_1$	27564.39
DrHD	5.50	27	28	17.00	61.94	–0.991	$C_{695}H_{1155}N_{223}O_{255}$	16715.16
DS+BnHD	8.51	33	31	20.85	55.27	–0.969	$C_{1011}H_{1659}N_{305}O_{358}S_1$	23847.18
BnHD	9.05	18	15	25.02	28.76	–1.487	$C_{536}H_{888}N_{168}O_{207}$	12997.95
DS+CeHD	5.01	104	126	8.30	44.28	–1.128	$C_{2588}H_{4096}N_{742}O_{954}S_4$	60997.63
CeHD	4.90	89	110	6.65	35.18	–1.294	$C_{2113}H_{3325}N_{605}O_{803}S_3$	50148.40
DS+YlHD	5.19	111	129	17.07	55.59	–1.030	$C_{3143}H_{4992}N_{904}O_{1103}S_1$	73123.67
YlHD	5.09	96	113	17.27	50.07	–1.150	$C_{2668}H_{4221}N_{767}O_{952}$	62274.43

Table S2. Secondary structure content in eight recombinant proteins was obtained by far UV CD spectrometry and calculated with CDNN software

Treatment	Protein	Random%	Antiparale%	Parallel%	Beta-Turn%	Helix%
Phosphate buffer	DS+DrHD	17.7	6.4	2.6	17.2	56.1
	DrHD	61.2	12.2	0.6	25.3	0.7
	DS+BnHD	25.5	2	4.6	14.2	53.7
	BnHD	66	7.6	0.5	25.2	0.7
	DS+CeHD	24.6	5.7	4	16.6	49.1
	CeHD	63.7	10.3	0.7	24.3	1
	DS+YlHD	16.7	1.8	2.6	14.3	64.6
	YlHD	81.5	0.3	0.6	14.8	2.8

Table S3 The characteristics of G3LEA proteins reported

Group	Protein	Species	Secondary Structures and Transitions	Methods	Reference
G3LEA	GmPM30	*Glycine max L.*	11.9% a-helix in solution; 56.8%, 63.5% and 81.4% a-helix with 4% SDS, 50% TFE, and 70% TFE , respectively	CD and FTIR	[57]
	D-7	*Typha latifolia*	Largely unstructured in solution; 24% β-sheet, 51% a-helix upon fast-drying, 45% β-sheet, 40% a-helix upon slow drying	FTIR	[37]
	COR15A COR15B	*Arabidopsis*	Both highly disordered in solution; 65% a-helix (COR15A) and 57% a-helix (COR15B) in the dry state	CD	[58]
	LEA7	*Arabidopsis*	Largely unstructured in solution; 15% β-sheet, 27% a-helix in a dry state, a-helix is promoted with lipid vesicles	FTIR and CD	[59]
	LEAM	*Pisum sativum*	Largely unstructured in solution; 50% a-helix with SDS, 70% a-helix with TFE or in a dry state	FTIR and CD	[22]
	AfrLEA2	*Artemia francis-cana*	4% a-helix in solution；24% a-helix with SDS and 41% a-helix with TFE; 46% a-helix upon drying	CD	[24]
	AfrLEA3m	*Artemia francis-cana*	2% a-helix in solution；41% a-helix with SDS and 36% a-helix with TFE;18% a-helix upon drying	CD	[24]
	AavLEA1	*Aphelenchus avenae*	Largely unstructured in solution; predominantly a-helical struc-tures with desiccation	FTIR and CD	[8]
	DrLEA3 (DosH)	*Deinococcus radiodurans*	45% α-helix formation under aqueous condition; Mn^{2+} or Zn^{2+} can increase the α-helix	CD	[25]
	MpLEA1	*Marchantia polymorpha*	Largely unstructured in solution; predominantly a-helical struc-tures upon drying	FTIR	[60]

Note: Circular dichroism (CD), differential scanning calorimetry (DSC), Fourier transform infrared spectroscopy (FTIR), and nu-clear magnetic resonance (NMR).

Table S4 Strains and plasmids used in this study

Strains/Plasmids	Description	Source
Plasmids		
pET28a（+）	*Kan^r^ oripBR322 lacl^q^ T7p*	Novagen
28a-*DS*	pET28a-derived plasmid carrying the *DS* gene	This study
28a-*DrHD*	pET28a-derived plasmid carrying the *DrHD* gene	This study

28a-*DS+DrHD*	pET28a-derived plasmid carrying the *Ds+DrHD* gene	This study
28a-*BnHD*	pET28a-derived plasmid carrying the *BnHD* gene	This study
28a-*DS+BnHD*	pET28a-derived plasmid carrying the *Ds+BnHD* gene	This study
28a-*CeHD*	pET28a-derived plasmid carrying the C*eHD* gene	This study
28a-*DS+CeHD*	pET28a-derived plasmid carrying the *Ds+CeHD* gene	This study
28a-*YlHD*	pET28a-derived plasmid carrying the *YlHD* gene	This study
28a-*DS+YlHD*	pET28a-derived plasmid carrying the *Ds+YlHD* gene	This study
Strains		
BL21-0	F^- *omp*T $hsdS_B(r_B^- m_B^-)$ *gal dcm*(DE3)	TransGen Biotech
BL21-pET	The control strain harboring the empty vector pET28a, Kan^r	This study
BL21-DS	The recombinant BL21 strain containing 28a-*DS*, Kan^r	This study
BL21-DrHD	The recombinant BL21 strain containing 28a-*DrHD*, Kan^r	This study
BL21-DS+DrHD	The recombinant BL21 strain containing 28a-*DS+DrHD*, Kan^r	This study
BL21-BnHD	The recombinant BL21 strain containing 28a-*BnHD*, Kan^r	This study
BL21-DS+BnHD	The recombinant BL21 strain containing 28a-*Ds+BnHD*, Kan^r	This study
BL21-CeHD	The recombinant BL21 strain containing 28a-*CeHD*, Kan^r	This study
BL21-DS+CeHD	The recombinant BL21 strain containing 28a-*Ds+CeHD*, Kan^r	This study
BL21-YlHD	The recombinant BL21 strain containing 28a-*YlHD*, Kan^r	This study
BL21-DS+YlHD	The recombinant BL21 strain containing 28a-*Ds+YlHD*, Kan^r	This study

Table S5 List of primers required for construction of recombinant *E.coli* strain

Gene name	Primer name	Sequence（5’-3’）	Fragment size
DS	EP 1	CAAATGGGTCGCGGATCCATGTTTGAACGCGATGAA	348 bp
	EP 2	CTCGAGTGCGGCCGCAAGCTTTTCCTTGCCCGCGTCC	
DS+DrHD	EP 3	CAAATGGGTCGCGGATCCATGTTTGAACGCGATGAA	832bp
	EP 4	CTCGAGTGCGGCCGCAAGCTTTCAGGTGCGCAGGTCGAAAGC	
DS (*BnHD*)	EP 5	Same as EP 1	347bp
	EP 6	TGTTGGGCCTTTTGAGCCGTTTCCTTGCCCGCGTCCTTGA	
BnHD	EP 7	TCAAGGACGCGGGCAAGGAAACGGCTCAAAAGGCCCAACA	404bp
	EP 8	CTCGAGTGCGGCCGCAAGCTTTCACGCTTCAGCCGTCTCTTTC	
DS (*CeHD*)	EP 9	Same as EP 1	347bp
	EP 10	TCCTTGGCACTGTTGTAAGCTTCCTTGCCCGCGTCCTTGA	
CeHD	EP 11	TCAAGGACGCGGGCAAGGAAGCTTACAACAGTGCCAAGGA	1472bp
	EP12	CTCGAGTGCGGCCGCAAGCTTTCAATCAGAAGCTTTTTCC	
DS (*YlHD*)	EP 13	Same as EP 1	347bp
	EP 14	TCAATGGCGTTCTCCTTGGCTTCCTTGCCCGCGTCCTTGA	
YlHD	EP 15	TCAAGGACGCGGGCAAGGAAGCCAAGGAGAACGCCATT	1766bp
	EP 16	CTCGAGTGCGGCCGCAAGCTTTCAAGTCTGGCCCAGCTCATTTG	

Note: GGATCC is the restriction enzyme site of BamH Ⅰ, and AAGCT is the restriction enzyme site of Hind Ⅲ. The brackets indicate that it is connected to this gene.

Experimental procedures

In silico analysis

Regions of protein disorder were predicted using the CSpritz web server (http://protein.bio.unipd.it/cspritz/) [61] and the IUPred (http://iupred.elte.hu/) [62]. The amino acid content and grand average hydropathy (GRAVY) values were estimated by the ProtParam tool (https://web.expasy.org/protparam/). A hydropathy plot was generated with the Kyte and Doolittle algorithm program (http://web.expasy.org/protscale/) [63].
